# A randomized controlled trial to prevent glycemic relapse in longitudinal diabetes care: Study protocol (NCT00362193)

**DOI:** 10.1186/1748-5908-1-24

**Published:** 2006-10-20

**Authors:** Mary Margaret Huizinga, Ayumi Shintani, Stephanie Michon, Anne Brown, Kathleen Wolff, Laurie Shackleford, Elaine Boswell King, Rebecca Pratt Gregory, Dianne Davis, Renee Stiles, Tebeb Gebretsadik, Kong Chen, Russell Rothman, James W Pichert, David Schlundt, Tom A Elasy

**Affiliations:** 1 Diabetes Research and Training Center, Vanderbilt University Medical Center, Nashville, TN, USA; 2 Division of General Internal Medicine and Public Health, Department of Medicine, Center for Health Services Research, Vanderbilt University Medical Center, Nashville, TN, USA; 3 VA Tennessee Valley Healthcare System, GRECC, Nashville, TN, USA; 4 Department of Biostatistics, Vanderbilt University Medical Center, Nashville, TN, USA; 5 School of Nursing, Vanderbilt University Medical Center, Nashville, TN, USA; 6 Division of Gastroenterology, Department of Medicine, Vanderbilt University Medical Center, Nashville, TN, USA; 7 Department of Biomedical Engineering, Vanderbilt University Medical Center, Nashville, TN, USA; 8 Department of Surgery, Vanderbilt University Medical Center, Nashville, TN, USA; 9 Center for Patient and Professional Advocacy, Vanderbilt University Medical Center, Nashville, TN, USA; 10 Energy Balance Laboratory, Vanderbilt University Medical Center, Nashville, TN, USA; 11 Department of Psychology, Vanderbilt University, Nashville, TN, USA; 12 VA National Quality Scholars Program, Nashville, TN, USA

## Abstract

**Background:**

Diabetes is a common disease with self-management a key aspect of care. Large prospective trials have shown that maintaining glycated hemoglobin less than 7% greatly reduces complications but translating this level of control into everyday clinical practice can be difficult. Intensive improvement programs are successful in attaining control in patients with type 2 diabetes, however, many patients experience glycemic relapse once returned to routine care. This early relapse is, in part, due to decreased adherence in self-management behaviors.

**Objective:**

This paper describes the design of the Glycemic Relapse Prevention study. The purpose of this study is to determine the optimal frequency of maintenance intervention needed to prevent glycemic relapse. The primary endpoint is glycemic relapse, which is defined as glycated hemoglobin greater than 8% and an increase of 1% from baseline.

**Methods:**

The intervention consists of telephonic contact by a nurse practitioner with a referral to a dietitian if indicated. This intervention was designed to provide early identification of self-care problems, understanding the rationale behind the self-care lapse and problem solve to find a negotiated solution. A total of 164 patients were randomized to routine care (least intensive), routine care with phone contact every three months (moderate intensity) or routine care with phone contact every month (most intensive).

**Conclusion:**

The baseline patient characteristics are similar across the treatment arms. Intervention fidelity analysis showed excellent reproducibility. This study will provide insight into the important but poorly understood area of glycemic relapse prevention.

## Background

Diabetes is a common disease and has great impact on the individual and society[1]. The burden of diabetes is expected to increase as the population ages, becomes more ethnically diverse and more obese[2]. Self-management of diabetes is critical to prevent the complications associated with diabetes and, yet, remains difficult for many patients to sustain.

Recent large randomized controlled trials have proven that tight glycemic control reduces the microvascular and macrovascular complications of diabetes [3-5]. Reduction of these complications also leads to a great cost savings to healthcare and society[6]. However, it has been difficult to translate the success of these large randomized control trials to everyday practice [7-9]. A recent cross-sectional analysis of 95 clinicians revealed only 40.5% of type 2 diabetes patients had a glycated hemoglobin (HbA1c) less than 7%[9]. Even large, well-conducted, multi-factorial randomized controlled trials aimed at reducing HbA1c have not had success in maintaining long-term glycemic control[10]. The disparity of care between the large trials and a primary care office is largely due to the difference in resources available in the typical medical office. Practical, sustainable ways of maintaining tight glycemic control are needed in everyday practice. Indeed, a number of for profit corporations have entered this arena of disease management given a seeming inability of the current clinical milieu to adequately address this issue.

While diabetes improvement programs are successful in acutely lowering HbA1c [11-24] the long-term effectiveness of these programs is disappointing. Approximately 40% of those who return to routine care after completing an intensive diabetes improvement program experience a relapse in their glycemic control within one year [25-27]. While some of the glycemic relapse may represent a natural progression of the underlying disease, it is unlikely that such a high percentage would experience such significant disease progression in such a short period of time[4,28]. Some proportion of the relapse is likely due to a patient's inability to maintain adherence to key self-care behaviors – diet, exercise, self-monitoring of blood glucose and medication regimen. Little is known about the optimal frequency, intensity or nature of maintenance interventions needed to prevent deterioration of self-care behaviors that lead to glycemic relapse.

## Hypothesis

The purpose of this study is to better understand prevention of glycemic relapse. The primary aim of this study is to assess the relative effectiveness of three management approaches, varying in frequency, for preventing glycemic relapse after glycemic control has been achieved through participation in an intensive diabetes improvement program. This study will determine the optimal frequency of intervention needed to prevent glycemic relapse in patients with type 2 diabetes. The authors hypothesize that high intensity intervention will lead to a decrease in glycemic relapse in a dose dependent fashion.

Other aims to be addressed in this study include determination of patient characteristics and behaviors predictive of glycemic relapse. In doing so, specific subgroups in need of alternative maintenance strategies will also be identified. Finally, this study will also determine the differences in activity cost between the intervention arms using activity based accounting.

## Methods

### Study Design

This study is a prospective, randomized control trial to assess the relative effectiveness of three management strategies for the purpose of preventing glycemic relapse in type 2 diabetes. The subjects will be randomized to one of three arms: routine follow-up in a primary care clinic (control), telephone contact every three months (moderate intensity) or telephone contact every month (high intensity). The duration of the study is 24 months. At the completion of the intervention period, the subjects will be asked to complete another 12 months of follow-up during which everyone will receive routine care only. The primary endpoint is glycemic relapse. Glycemic relapse is defined as a HbA1c greater than 8% and an increase by 1% point from baseline. The primary analysis will be based on intention to treat.

### Study Setting

Telephonic intervention based out of an academic center in middle Tennessee. At recruitment, study participants lived in the city and surrounding suburbs of the academic center.

### Study Population

All subjects are recruited after completion of a 12 week outpatient, intensive diabetes improvement program following referral for poor glycemic control (HbA1c>8%). The intensive improvement program consists of instruction and support in diabetes self-management coupled with intensification of glycemic medications, including insulin. It is provided by nurse practitioners and supervised by a practicing diabetologist. The educational content includes diet, exercise, self-monitoring of blood glucose and medication adherence as well as instruction in preventive measures such as foot care and screening for complications. Upon completion of the program, only those subjects referred to the improvement program for poor glycemic control (HbA1c>8%) and who obtained control (HbA1c<8% and at least an absolute 1% decline in HbA1c) during the program were recruited. Only subjects aged 18–75 years of age were included. Pregnant women were excluded.

### Randomization

**Figure 1:**
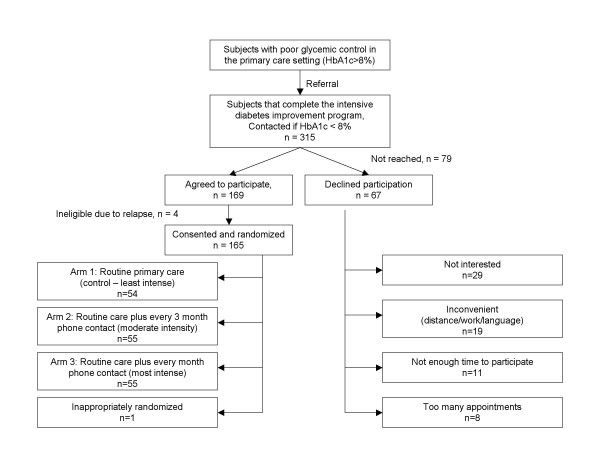
Enrollment and Randomization.

Two weeks after completion of the improvement program, a research assistant contacted patients and gave them a brief explanation of the study. The subjects were then invited to participate in the study if they met the defined inclusion criteria. A research assistant confirmed eligibility. After informed consent was obtained, patients were randomly assigned to one of three study arms. Randomization applied permuted block scheme for balancing interval, varying randomly among 3, 6, 9 and12 according to the outcome of a computer generated random number. This ensured that the cumulative number of assignments to each treatment would be in balance after each block of assignments had been made. The allocation sequence was written by the statistician involved with the trial. Once treatment arm status was assigned by the research assistant, subjects in the intervention arms were assigned a study nurse practitioner. Due to the nature of this intervention, blinding of participants, investigators and study nurse practitioners was not possible. See Figure 1 for enrollment and randomization scheme.

### Intervention

**Figure 2:**
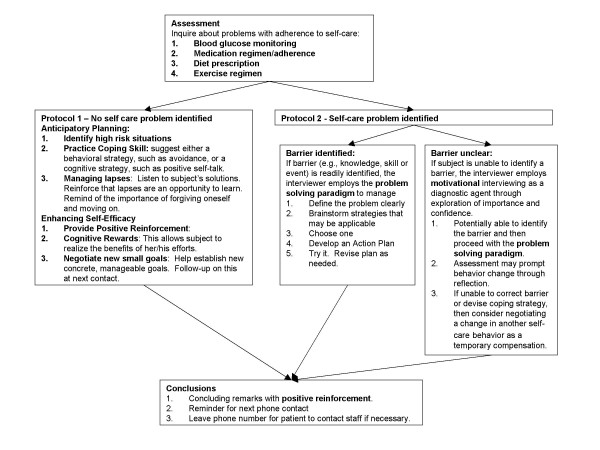
Telephone Contact Intervention Flow Sheet.

The intervention consists of a phone contact by a nurse practitioner with a referral to a dietitian if nutrition self-care is perturbed. The characteristics of the intervention are described in Table 1 using a diabetes intervention taxonomy previously characterized[29] The duration of each contact was monitored. During the first session, shared goal setting was established and referred to or modified during subsequent contacts. The method and content of the phone contacts varied based on the assessment. If there were no problems related to glycemic control or self-care behaviors identified, then Protocol 1 was followed (see Figure 2). If a problem was identified, Protocol 2 was followed (see Figure 2). The intervention does not vary between the treatment arms; only the frequency of the intervention varies.

**Table 1:** Intervention Structure

Setting	One-on-One
Delivery	Phone contact
Teaching Methods	Shared goal setting Problem solving Cognitive re-framing Diaries
Content	Diet Exercise Self-monitoring of blood glucose Medication management
Provider	Diabetes certified nurse educator with a dietician referral if diet self-care is perturbed
Tailoring of intervention to an assessment	Yes
Modification of intervention with follow-up	Yes
Intensity of intervention	
Number of episodes	Arm 2: 8 Arm 3: 24
Duration of episodes	Measured as part of study protocol
Duration of study	24 months
Initial supplement given	No

Protocol 1 is characterized by anticipatory planning for potential lapses, including practicing a coping skill, and also offers self-efficacy enhancement through positive reinforcement, short-term goal setting and cognitive rewards. If a self-care problem was identified then protocol 2 was followed. The subject was asked to identify the source of the struggle. If readily identified, the interviewer employed a 5 step problem solving paradigm: 1) Define problem clearly, 2) Brainstorm strategies, 3) Choose a strategy, 4) Develop an action plan and 5) Try it and revise as needed. If a subject was unable to identify a reason for deteriorating self-care behavior, motivational interviewing was employed largely as a diagnostic modality[30] Subjects were asked to assess the importance of and their confidence in correcting the lapse behavior. The individual was then asked to comment on what prevented them from giving a higher importance/confidence score. This often identified an underlying problem which led back to the problem solving paradigm. In addition to providing a diagnostic tool for identification of the reasons behind the lapse, the motivational interview may also provide a cue to action via the subject's reflection during the assessment.

The interviewer worked with the subject to correct (e.g. correcting a cognitive distortion) the underlying reason for the perturbation of the self-care behavior. If the obstacle could not be corrected (i.e. divorce, financial barrier), then the interviewer worked with the subject to develop a coping mechanism. However, if the subject remained unable to identify a reason for lapse in self-care behavior or to devise a coping strategy, the interviewer worked with the subject to negotiate a change in another self-care behavior as a compensation for the perturbed behavior. A negotiated compensation, for example, may include increased exercise, increased monitoring or increased insulin use for a perturbation of diet self-care.

### Intervention Fidelity

To enhance the reliability and validity of the behavioral intervention portion of this study, intervention fidelity tools were used to monitor the phone contacts between the nurse practitioners and the study subjects [31-33]. The analysis consisted of qualitative descriptions of the extent to which a sample of intervention phone calls was consistent with the intervention protocol (Figure 2) and guidelines. Consistency between the nurse practitioners was also determined. Raters used checklists derived from the protocols to document which elements were conducted or omitted. Overall, adherence to the protocol was quite high with almost all elements present in more than 80% of all interviews. The educators did not differ significantly in any category.

### Primary Outcome

The primary outcome is the glycemic relapse rate at 24 months. Relapse is defined as a HbA1c greater than 8% and an absolute 1% increase from baseline. The HbA1c will be measured at baseline and at 6-month intervals throughout the study.

### Secondary Outcomes

#### Activity Assessment

A pager-sized (2.8 × 2.2 × 1.1 inches, weighing 2.3 oz) triaxial portable accelerometer (RT3 Research Activity Tracker by StayHealthy, Inc. Monrovia CA) is used to measure detailed movements in the center of body mass (worn at the hip). The RT3 monitor is programmed with each study participant's weight, height, age, and gender prior to application. During each of the visits, each subject is fitted with the RT3 monitor securely on his/her right hip, either by direct clipping to the belt or using a small pouch-bag (for women who do not usually wear a waist belt). Subjects are instructed to wear the RT3 monitor during all possible non-sleeping activities, except during water sports, for the next 7 days. Once the monitor is initialized, it runs continuously without interruption from the subject (no buttons to push). At the end of the 7-day monitoring period, the RT3 is mailed back to the study coordinator via a pre-addressed/stamped bubble envelope and its data downloaded. Using the raw activity counts and a prediction model which was previously developed and validated[34], the total energy expenditure and overall physical activity levels during each study period are obtained for each subject. Furthermore, utilizing durations of activities within certain intensity categories (utilizing the minute-to-minute measurements), subject's adherence to exercise will be validated.

#### Depression Score

The Center for Epidemiologic Studies Depression Scale (CES-D) is used to assess depression in this study. The CES-D is a well-validated, 20 item self-administered questionnaire that quantifies the frequency of depressive symptoms over the previous 7 days. Four items are reversed scored and the total possible score is 60 with 0–9 representing no to minimal symptoms, 10–16 mild symptoms, 17–24 moderate symptoms and >24 severe depressive symptoms.[35]

#### Cost Accounting Analysis

Cost analysis of the interventions will be assessed using activity based cost (ABC) accounting techniques[36]. ABC differs from conventional cost accounting in that ABC establishes a causal relationship between work performed, the costs thereof, and the clinical outcomes of the same. In so doing, ABC enables researchers to quantify more precisely the costs of interventions, the skill level of the team member performing the task, the sequence of activities, and the patients' outcomes.

### Data Management

Data is entered into MS Access (Microsoft Corporation, Redmond, WA) tables. Management report generating programs are used to track subject's progress through the study and to generate letters when visits are due. This also allows for early identification of missing data.

### Study size

Sample size calculation was performed based on chi-square test for linear trend in proportions of patients among the three study arms who relapsed during the study period (118). We expected 50% of patients who are assigned to study arm A (routine primary care follow-up) relapse during the study period, 30% in the study arm B (scheduled 3 month interaction with a certified nurse), and 20% in the study arm C (scheduled 1 month interaction with a certified nurse). Anticipating 20% attrition, 165 subjects (55 recruited/44 complete study) will provide 85% power to detect statistically significant linear trend at 2-sided 5% alpha level. Calculations for power analysis were performed by using nQuery Advisor version 4.0 (Statistical Solutions, Stonehill Corporate Center, Saugus, MA).

### Ethics

This trial received approval from the Vanderbilt Institutional Review Board. An information sheet was given to all subjects and those who agreed to participate were consented prior to randomization. Informed consent was obtained from all subjects. Subjects are free to withdraw from the study at any time, although they were encouraged to decline randomization unless they were prepared to participate in the study for 24 months. The confidentiality of the study data are maintained as follows: once computerized, data are not linked to identifying information and the original documents are kept in locked cabinets. The computerized records are identified by study number which is the only link to the subject's identification. Access to the identifying information is restricted to the principal investigator and the study coordinator. Patients received $50 upon completion of the study.

## Population characteristics

Enrollment started June 2002 and concluded in January 2005. A total of 164 subjects completed randomization. The control group consists of 54 subjects and each of the intervention arms consists of 55 patients. The baseline characteristic were similar across the groups, see Table 2, with no statistically significant differences.

The average age (± SD) of the population was 55 ± 10.7 years. Forty-four percent were female and 20% were African-American. The average HbA1c (± SD) was 6.7 ± 0.68 and the average duration of diabetes (± SD) was 7.1 ± 8.2 years. Fifty-four percent used insulin with a median of 55 (IQR 25–92) units/day of insulin. The average BMI (± SD) was 34 ± 6.9 kg/m^2 ^and the average waist circumference (± SD) was 42.9 ± 5.8 cm. Results for the CES-D were available for 118 subjects and the median CES-D was 9 (IQR 4–17). The CES-D results were available with equal frequencies in each study arm.

Baseline physical activity data was successfully obtained in 154 subjects. The baseline measures of daily energy expenditure, physical activity level (PAL) and time spent in moderate and vigorous physical activities (MVPA) were similar in all three groups (see Table 2) and fairly similar to average sedentary populations.

The initial nurse's assessment for the intervention groups were similar (see Table 3). The initial assessment occurred within 2 months of the completion of the intensive outpatient diabetes improvement program. The average number of minutes spent on the initial phone contact was 19.6 ± 9.3. Five variables were assessed by the nurses including glycemic control, self blood glucose monitoring, medication adherence, diet adherence and exercise adherence. The majority of the patients answered unchanged in each category for this baseline assessment. At baseline, 28% had already self-reported worsening of their glycemic control since completion of the improvement program.

**Table 2:** Baseline population characteristics

Characteristic	Control Group (n = 54)	Moderate Intensity Group (n = 55)	High Intensity Group (n = 55)
Age, yrs	56.2 ± 10	55.7 ± 11	53.5 ± 11
Female, n (%)	23 (43)	21 (38)	28 (51)
African American, n (%)	7 (13)	16 (29)	12 (22)
≥ High School, n (%)	47 (87)	49 (89)	50 (91)
Duration of diabetes, yrs	5.5 (0.7–10)	4.0 (0.5–10)	4.0 (0.5–10)
Insulin use, n (%)	32 (54)	49 (45)	50 (58)
Units of insulin per day	39 (24–79)	59 (32–100)	61 (25–93)
Weight, lbs	225 ± 48	215 ± 37	223 ± 51
BMI	34 ± 7	33 ± 6	35 ± 7
Waist circumference, in	43.5 ± 6.2	41.8 ± 4.8	43.3 ± 6.3
HbA1c	6.7 ± 0.7	6.6 ± 0.7	6.8 ± 0.6
Systolic BP	126 ± 15	125 ± 17	127 ± 15
Diastolic BP	72 ± 9	72 ± 11	73 ± 12
Total cholesterol	177 ± 28	178 ± 35	174 ± 34
HDL	43 ± 13	44 ± 11	41 ± 11
LDL	97 ± 28	97 ± 30	98 ± 31
Triglycerides	185 (124–229)	168 (124–246)	161 (112–219)
CES-D	9 (4–18)	10 (4–17)	7 (4–14)
DEE	3007 ± 671	2963 ± 659	3097 ± 869
PAL	1.31 ± 0.08	1.32 ± 0.08	1.34 ± 0.09
MVPA	62 (35–91)	61 (40–116)	77 (41–126)

Reported as mean ± standard deviation or median (interquartile range).

n – number; BMI – body mass index (kg/m^2^); HbA1c – glycated hemoglobin (%); BP – blood pressure; HDL – high-density lipoprotein (mg/dL); LDL – low-density lipoprotein (mg/dL); DEE – daily energy expenditure (kcal); PAL – physical activity level = total energy expenditure/resting energy expenditure; MVPA – moderate to vigorous physical activity (intensity >3 × resting energy expenditure) (min/day)

## Discussion

This study will advance our understanding of maintenance of glycemic control. The authors approached relapse prevention in a novel way – by determining the "dose" of intervention needed to prevent glycemic relapse. The intervention is carefully outlined to allow for reproducibility. Intervention fidelity is excellent. This study will also compare the cost of the intervention to routine care. As there is a burgeoning business in chronic care management, it is important to study chronic care interventions for both efficacy and cost-effectiveness to aid in the development of evidence based services.

While little is known about relapse of glycemic control, extrapolation is possible from the practical experience available in the obesity, alcohol and smoking literature. Perri et al demonstrated that routine contact with providers was the only variable predictive of weight loss maintenance[37]. Baum et al found that a 3 month provider supported program resulted in greater maintenance of initial weight loss for 12 months as compared to a control group[38]. To minimize relapse after alcohol treatment, Marlatt recommends a behavioral maintenance package consisting of identification of high-risk situations, training in problem solving, actual practice coping with high-risk situations and development of cognitive coping skills[39]. Baer's cognitive behavior model of the relapse process in smoking puts forth that due to prior poor conditioning, individuals are actively coping with situation specific urges to smoke[40]. To prevent smoking relapse, Baer recommends systematic but brief assessment, encouragement, goal setting, planning for risk, reinterpreting lapses, recommendations for lifestyle changes and follow-up appointments. The study intervention is firmly rooted in health behavior methods and draws from prior experience in other diseases such as obesity, smoking and alcohol. While maintenance of self-care behaviors is critical to prevent glycemic relapse, the "dose" of maintenance intervention needed is unknown.

Limitations of this study include reproducibility of the intervention and the possible differences in the routine care received. While the intervention is outlined in this article, it may be difficult to reproduce the problem solving skills used by the nurse practitioners in this study for someone with no prior training. The frequency of the intervention is varied but not the intervention content – it is possible that another intervention would be more effective. This study was not designed to compare effectiveness of different interventions, but to determine the optimal frequency of an intervention that was thought to be optimal based on a previously published meta-analysis[41]. The study protocol did not address how often the subjects saw their primary care providers, the care provided by the primary care providers or counseling given in that setting.

This study seeks to assess the efficacy of varying frequencies of a highly structured nurse initiated telephonic intervention for the prevention of glycemic relapse. Prevention of glycemic relapse is a novel area in diabetes care that remains largely unstudied. By adjusting the frequency of the intervention, the optimal "dose" of intervention to maintain adequate glycemic control can be determined. This study will add to the fund of knowledge on longitudinal diabetes care.

## Competing interests

The author(s) declare that they have no competing interests.

## Authors' contributions

MMH participated in the statistical analysis and was the primary writer of the manuscript. AS participated in the study design and performed the statistical analysis. SM and LS assisted with study implementation, data acquisition and database management. AB, KW, EBK, RPG and DD assisted with study implementation and data acquisition. RS participated in study design and will perform economical analysis. TG assisted with statistical analysis. KC participated in study design, physical activity data and analysis of the physical activity data. RR, DS participated in study design. JWP assisted with study design and performed the intervention fidelity analysis. TAE conceived of the study, participated in the design, analysis, data management and helped draft the manuscript. All authors read the manuscript, provided editorial comments and approved the final manuscript.

**Table 3:** Baseline nurses' assessment

Variable	Moderate Intensity Group (n = 55)	High Intensity Group (n = 55)
Length of phone call, min	21.0 ± 9.4	18.7 ± 8.9
Glycemic control		
Improved	12 (22)	10 (19)
Unchanged	30 (56)	25 (48)
Worse	12 (22)	17 (33)
Self blood glucose monitoring		
Improved	3 (6)	1 (2)
Unchanged	43 (81)	39 (74)
Worse	7 (13)	13 (25)
Medication adherence		
Improved	1 (2)	1 (2)
Unchanged	46 (87)	45 (85)
Worse	6 (11)	7 (13)
Diet adherence		
Improved	9 (18)	8 (15)
Unchanged	33 (65)	34 (64)
Worse	9 (18)	11 (21)
Exercise adherence		
Improved	12 (23)	14 (27)
Unchanged	23 (44)	26 (50)
Worse	17 (33)	12 (23)

Reported as mean ± standard deviation or n (%).
